# Skin-on-a-Chip Device for Ex Vivo Monitoring of Transdermal Delivery of Drugs—Design, Fabrication, and Testing

**DOI:** 10.3390/pharmaceutics11090445

**Published:** 2019-09-02

**Authors:** Bence Lukács, Ágnes Bajza, Dorottya Kocsis, Attila Csorba, István Antal, Kristóf Iván, András József Laki, Franciska Erdő

**Affiliations:** 1 Faculty of Information Technology and Bionics, Pázmány Péter Catholic University, Práter u. 50a, H-1083 Budapest, Hungary; 2 Biological Research Center, Hungarian Academy of Sciences, Temesvári krt. 62, H-6726 Szeged, Hungary; 3 Department of Pharmaceutics, Semmelweis University, Hőgyes Endre u. 7, H-1092 Budapest, Hungary; 4 Department of Biophysics and Radiation Biology, Semmelweis University, Tűzoltó u. 37-47. H-1094 Budapest, Hungary

**Keywords:** skin-on-a-chip, microfluidics, Franz diffusion cell, transdermal microdialysis, drug delivery

## Abstract

To develop proper drug formulations and to optimize the delivery of their active ingredients through the dermal barrier, the Franz diffusion cell system is the most widely used in vitro/ex vivo technique. However, different providers and manufacturers make various types of this equipment (horizontal, vertical, static, flow-through, smaller and larger chambers, etc.) with high variability and not fully comparable and consistent data. Furthermore, a high amount of test drug formulations and large size of diffusion skin surface and membranes are important requirements for the application of these methods. The aim of our study was to develop a novel Microfluidic Diffusion Chamber device and compare it with the traditional techniques. Here the design, fabrication, and a pilot testing of a microfluidic skin-on-a chip device are described. Based on this chip, further developments can also be implemented for industrial purposes to assist the characterization and optimization of drug formulations, dermal pharmacokinetics, and pharmacodynamic studies. The advantages of our device, beside the low costs, are the small drug and skin consumption, low sample volumes, dynamic arrangement with continuous flow mimicking the dermal circulation, as well as rapid and reproducible results.

## 1. Introduction

For testing drug absorption across the dermal barrier there are in vitro, ex vivo, and in vivo techniques in the toolbox of pharmaceutical researchers. For in vitro testing, different types of membranes are used and mounted in a gold-standard system of horizontal or vertical Franz diffusion cells as a first approach [1]. Usually, polymeric membranes [2,3] are applied for release testing. According to the Food and Drug Administration (FDA) SUPAC-SS (Scale-Up and Post Approval Change Semisolids) (May 1997), inert and synthetic membranes such as polysulfone and cellulose acetate/nitrate mixed ester can be used. Generally, hydrophilic polymeric membranes with a pore size of 0.45 μm are used. Based on our previous results, this pore size is equivalent to the porosity of the 10-tape-stripped rat skin [4]. The next step in the complexity of the drug release and penetration testing is the application of different skin preparations from animals and humans in Franz cells. The small animal (such as rat, mouse, and rabbit) skins [4] have traditionally been used in studies to predict human tissue permeability as they are usually inexpensive to purchase and maintain. Those tissues are thinner than human skin and have different morphologies resulting in higher compound permeabilities. Large animal skins (such as monkey, dog, and pig) [5,6,7] have also been used extensively but are expensive to purchase, especially in the cases of monkeys and dogs. Pig and monkey soft tissue is very similar to human soft tissue in terms of morphology and function; therefore, they are widely used as a substitute for human skin. The ex vivo human tissue [8] is difficult to obtain in sufficient quantities from normal, healthy subjects to perform permeability experiments with large enough sample sizes for statistical analysis. Many laboratories use, therefore, human cadaver skin [3,9] obtained from accredited tissue banks. However, ethical and legal concerns may arise when obtaining human tissue biopsies and surgical specimens. 

To obtain predictive and real-time results considering the kinetics and dynamics of drug delivery into the skin, in vivo transdermal microdialysis can be a good choice in different species. Both the ex vivo and the in vivo techniques have strengths and weaknesses. The strengths are that they are widely used in the drug development and pharmaceutical research and provide reliable and reproducible results. The disadvantages are the robustness, the high tissue, test compound and formulation needs, and the high costs. The usual size of adsorption surface in Franz diffusion cells is ~1.3–1.8 cm^2^/chamber and 6 parallels are simultaneously studied. To set up the instrument, several animals need to be sacrificed or human tissue should be purchased from plastic surgery or other sources. For use of human tissue ethical permissions are needed. To overcome these difficulties the miniaturization of Franz diffusion cell systems looks a promising option.

Skin-on-chip devices offer innovative and state-of-the-art platforms essential to overcome the above-mentioned limitations of Franz diffusion cells [10]. In some studies, organotypic cultures are cultivated in the chips to mimic human skin and the transepithelial electrical resistance (TEER) is detected [11,12,13] for characterization the diffusion properties. The tissue vascularization can also be considered in the preparation of skin cell containing co-cultures [14]. The impact of tissue engineering, biomaterials, and microfluidic platforms on the development of skin grafts and biomimetic in vitro skin models are reviewed by Mohammadi and co-workers [15] and these techniques can also be used for the development of novel skin disease platforms for drug-screening assays.

In our laboratory, a novel, temperature-controlled microfluidic device has been designed and fabricated for testing drug penetration through the skin. To evaluate its function under real conditions, a hydrophilic model drug, caffeine, has been tested in a Vaseline-based cream formulation. The drug penetration has been measured in native and sensitized mouse and rat skin in the skin-on-a chip device and for comparison and evaluation Franz diffusion cell and transdermal microdialysis experiments were also performed.

## 2. Materials and Methods

### 2.1. Materials and Solutions

Both for in vivo and for ex vivo experiments, peripheral perfusion fluid (PPF) was used as a physiological solution. Chemical composition of PPF: 147 mM NaCl, 4 mM KCl, and 2.3 mM CaCl_2_·2 H_2_O. All substances were purchased from: Sigma-Hungary Kft, Budapest, Hungary.

### 2.2. Cream Formulation

A cream containing 2% of caffeine was used in all studies. Caffeine was acquired from Sigma-Hungary Kft (Budapest, Hungary). 

The test formulation of caffeine was compounded according to the following description: 2 g of caffeine was dispersed with 4.1 g of liquid paraffin (Ph. Hg.) in a mortar with pestle, then 47 g of white soft paraffin ointment (containing polysorbate 60 (Ph. Hg.) 4 m/m%, liquid paraffin 8 m/m%, white soft paraffin 26 m/m%, cetostearyl alcohol (Ph. Hg.) 12 m/m%, propylene glycol 10 m/m% and purified water 40 m/m%), 10 g propylene glycol (Ph. Hg.), and 36.9 g of 0.21 m/m% citric acid aqueous solution were added. 

### 2.3. Franz Diffusion Cell Study

The Franz diffusion cell system (Hanson, ABL&E-JASCO Hungary Kft, Budapest, Hungary) used in the study is composed of six sampling units with vertical arrangement. Every cell has a donor and a receptor chamber, the latter contains PPF, its capacity is 7.5 mL. The test skin preparation, which has the test drug in proper quantity (0.5 g) on its external surface was placed between the two compartments. Attention was paid to the airless placement of the skin. 

The drug penetrates through the skin into the PPF. In the acceptor unit a helical mixer is applied to generate continuous stirring, which can stimulate the drug release and ensures the homogeneity of the fluid. The release medium was mixed on 320 rpm and heated at 32 °C. 

#### 2.3.1. Skin Preparation

Male Wistar rats (ToxiCoop, Budapest, Hungary) weighing 300–340 g and male NMRI mice (ToxiCoop, Budapest, Hungary) of 30–40 g were used for the experiments. Chloralhydrate (purchased from Sigma-Hungary Kft, Budapest, Hungary) was applied as anesthetics in a dose of 400 mg/kg i.p. in rats and 450 mg/kg i.p. in case of mice. 

Initially, the hair on the abdominal skin was removed; the skin was shaved off and wiped dry. An emphasis was put on the tape-stripping method. The goal was to study the penetration blocking effect of the stratum corneum layer, therefore one half of the skin preparations was tape-stripped 10 times. The process resulted in the partial removal of dead keratinocytes. The skin surface treated with caffeine cream was 1.766 cm^2^, accordingly the dose of applied caffeine was 6.0 mg/cm^2^.

The skin preparations were stored in PPF on room temperature until the initiation of ex vivo experiments. The Franz diffusion cell studies were started within 12 h after the surgery. At the beginning of the experiments the preparations were placed on the cells. The stratum corneum layer was facing the donor chamber, contacting the test drug and the dermal-subcutaneous surface was contacting the PPF on the acceptor compartment’s side.

#### 2.3.2. Sample Collection

Samples have been collected from the receptor chamber through a needle in every 30 min for six hours. Peripheral Perfusion Fluid 0.5 mL of volume was injected in the receptor compartment through a replacement port at every sampling occasion. That resulted in the outflow of 0.5 mL sample through the sampling needle.

### 2.4. Transdermal Microdialysis Study

Microdialysis is an in vivo sampling technique for monitoring the biochemical changes of the extracellular milieu in different tissues with minimal tissue damage [16]. In vivo transdermal microdialysis was performed according to our previous paper [17]. Briefly, one or two microdialysis probes with a semipermeable membrane were introduced into the dermal layer of the skin, and were continuously perfused with the peripheral perfusion fluid (PPF). Samples were collected into vials and analyzed.

#### 2.4.1. Animals

Adult male Wistar rats (ToxiCoop Zrt., Budapest, Hungary), weighing 280 to 330 g, and adult male NMRI mice (ToxiCoop Zrt., Budapest, Hungary) weighing 30 to 35 g, housed in animal room (12 h light/dark periods, 22 ± 3 °C temperature, 50 ± 20% humidity) fed with commercial laboratory chow and tap water *ad libitum*, were used. All experiments in this study were conform to the guidelines of the Association for Assessment and Accreditation of Laboratory Animal Care International’s expectations for animal use, and were licensed by the Directorate for the Safety of the Food Chain and Animal Health, Budapest, and Pest County Agricultural Administrative Authority, Hungary (PE/EA/672-6/2016, 8 April 2016).

#### 2.4.2. Surgery and Performance of Microdialysis Experiments

Preparation of both rat and mouse abdominal skin surface was performed in the in vivo laboratory, on the day before the microdialysis experiments. Animals were anaesthetized with chloral hydrate (Sigma-Aldrich, Steinheim, Germany), 400 mg/kg i.p. for rats, and 450 mg/kg i.p. for mice. The abdominal skin surface was shaved off with an electric shaver, then epilated by an epilator cream (ISANA^®^ cream from Rossmann, Burgwedel, Germany), after that the animals returned to their cages.

Next day, on the day of transdermal microdialysis experiment, animals were reanesthetized intraperitoneally with chloral hydrate and fixed in supine position on a heating pad to keep their body temperature at a constant of 37 °C during the in vivo experiment. The abdominal skin was wiped dry. Rats and mice were randomly divided into two groups; the skin of five rats and five mice were sensitized 10 times (10 TS) by tape-stripping with leucoplast (BSN Medical GmbH, Hamburg, Germany) to remove the dead cell layer of stratum corneum; the skin of another five rats and five mice were not tape-stripped (0 TS).

**Figure 1:**
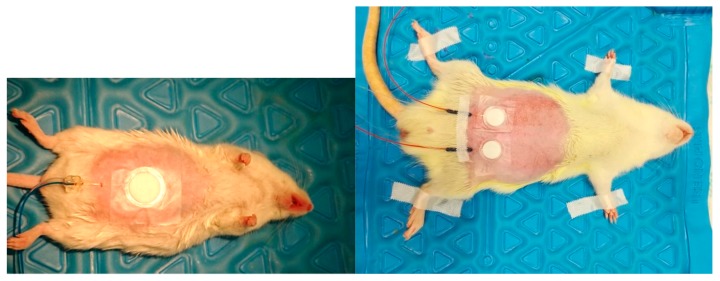
Implantation of microdialysis probes and placement of drug-containing patches in a representative mouse (left) and rat (right) in transdermal microdialysis experiments.

After the sensitization, the microdialysis probes were introduced through a guide needle (18G) into the dermal tissue. The guide needle was then withdrawn, and the probe membrane stayed in the dermal tissue. In rats two probes (MAB 11.8.10, Microbiotech, Stockholm, Sweden) were introduced, in parallel, 20 mm distance to each other; in mice one single microdialysis probe (MAB 7.8.10, Microbiotech, Stockholm, Sweden) was inserted (Figure 1). Probes were then fixed carefully with a tape (Omnisilk, Hartmann, Germany). The position of the probes was verified in a pilot experiment by ultrasound scanning. The microdialysis probes were continuously perfused with artificial PPF at 1 μL/min flow rate. 

Thereafter, transdermal patches (Curatest^®^F, Lohmann Rauscher GmbH, Rengsdorf, Germany), containing 50 mg of 2% caffeine cream formulation were placed on the skin, exactly above the position of microdialysis probe membrane.

#### 2.4.3. Sample Collection

Dialysate samples were collected into 300 μL collection vials every 30 min. One sample collection was performed before the placement of the patches (for equilibration), dialysis sampling was then maintained for a total of 4 h in mice, and 5 h in rats. Collected dialysate samples were immediately placed on dry ice, and samples were stored at −80 °C in a freezer until bioanalysis.

### 2.5. Skin-on-a-Chip Diffusion Cell Study

#### 2.5.1. Design and Fabrication of the Device

**Figure 2:**
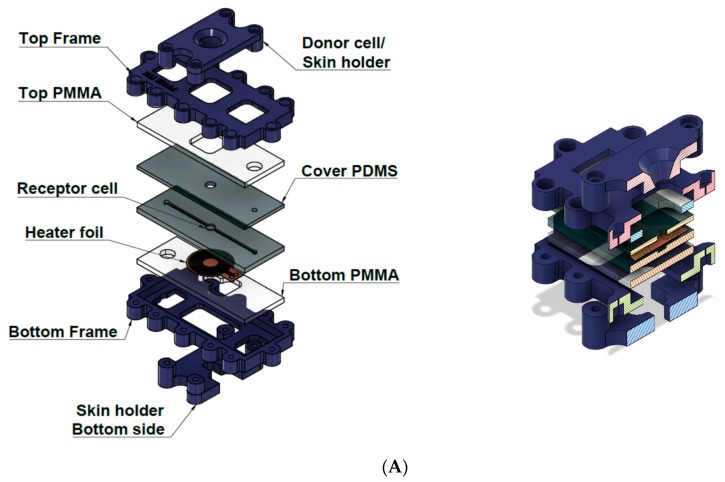

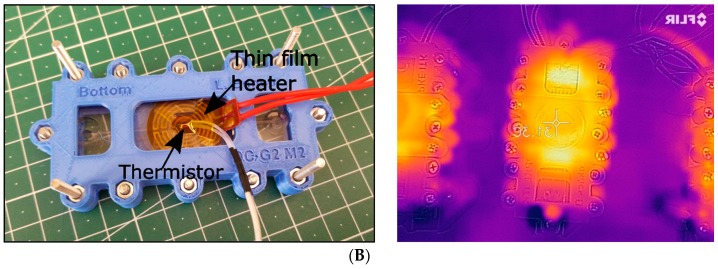
Exploded schematic view of the Microfluidic Diffusion Chamber (MDC) (**A**). Left: Layer-by-layer view of the device. Right: Cross sectional view of the device. Temperature-controlled skin-on-a chip device (MDC) (**B**). Left: The bottom side of the chip with the circular thin film heater and thermistor. Right: The temperature control measurement with a thermo-camera.

We have developed a Microfluidic Diffusion Chamber (MDC) (Figure 2A) for in vitro/ex vivo monitoring of transdermal delivery of topical drugs. In the current study caffeine was used as a hydrophilic model drug. The microfluidic channel system is fabricated by polymer-based techniques, which has been surrounded by poly(methyl methacrylate) (PMMA) and polylactic acid (PLA) components (Sunlu, Zhuhai, China) using Crafbot 2 (Creafbot Unique, Budapest, Hungary) and a FFF (Fused Filament Fabrication) type 3D printer. The MDC device had a temperature-controlled module, which was devoted to set the process at constant temperature (Figure 2B). The embedded heating element was automatically regulated by Arduino UNO (Arduino, Somerville, MA, USA) via a built in proportional–integral–derivative (PID) controller function. The device is made of three functional elements: on top is a donor compartment where the examined cream is poured, in the middle is an integrated skin sample (or alternatively a membrane), and the bottom is the receptor compartment. A heating foil was placed between the bottom acrylic glass and the bottom polydimethylsiloxane (PDMS) layer exactly under the receptor cell to maximize the heat transfer and minimize the power consumption. The donor compartment has a truncated cone shape, where top surface radius is 18 mm, the base radius is 8 mm and the height is 6 mm, and this fixes the skin sample onto the microfluidic structure. The receptor compartment is implemented into the microfluidic channel system, which is made of PDMS (SYLGARD™ 184 Silicone Elastomer Kit, Dow Consumer Solutions, Los Angeles, CA, USA). The microfluidic channel is 1.4 mm wide, 1 mm high, and 60 mm long, and has one inlet and one outlet. It connects to the receptor compartment with 5 mm diameter in the middle. PDMS liquid monomer and its treating agent are mixed at a 10:1 ratio, and incubated at 70 °C for 2 h. The polymerized PDMS is manufactured into the proper shape and punched through for 1 mm diameter of inlet, 1 mm diameter of outlet and 5 mm diameter of reservoir (receptor chamber). The temperature was transferred by flexible electric polyimide film heater and controlled by a thermostat (TSM 125 Temperature controller, H-Tronic, Hischau, Germany).

#### 2.5.2. Lab-on-a-Chip Sample Collection

**Figure 3:**
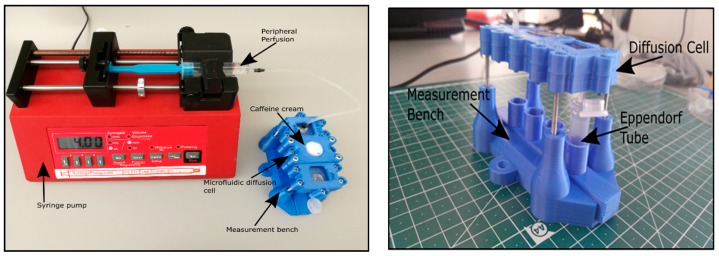
The performance of a microfluidic diffusion chamber (MDC) experiment. Left: The set-up of the microfluidic system with a syringe pump and the chip. Right: The position of the sample collection vial in the 3D printed measurement bench of the microfluidic device.

The PPF solution has been loaded into a 5 mL syringe. The air bubbles have been removed previously from the syringe and from the connected Teflon tubing before the load. The backpressure has been generated by a programmable syringe pump (NE-1000, New Era, Farmingdale, NY, USA). The flow rate has been set at along the entire measurement. The PPF solution has been forced through the microfluidic system, filled the receptor chamber and left on the outlet into collection vials (Figure 3). 

### 2.6. Bioanalysis

#### 2.6.1. Spectroscopy-Franz Diffusion Cell Samples

The caffeine content of the Franz cell samples was measured with a Shimadzu UV-visible spectrophotometer UV-1650PC. The collected samples were diluted with 1.5 mL PPF, thereby the cuvettes were filled with 2 mL of diluted sample. The concentration was measured against the buffer. The absorbance was accepted between 0.1 and 1.1, otherwise the samples were further diluted.

The absorption maximum of the caffeine was detected at 273 nm. From these values, caffeine concentrations were calculated according to the Lambert-Beer law:

*E* = log(*I*/*I*_0_) = ε*cL*,

where *E* means the absorbance, *I*_0_ = light intensity in front of the cuvette, *I* = light intensity behind the cuvette, ε = molar extinction coefficient, *c* = concentration and *L* = thickness of the cuvette.

#### 2.6.2. LC-MS/MS-Dialysate and Chip Samples

**Figure 4:**
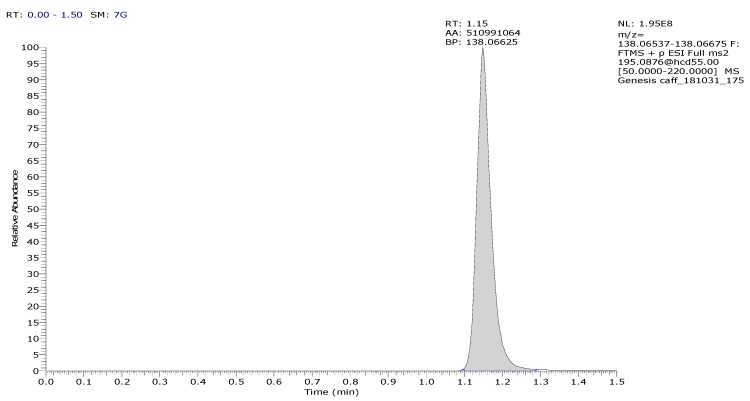
Typical chromatogram of caffeine with the method described in the LC-MS/MS method section.

**Figure 5:**
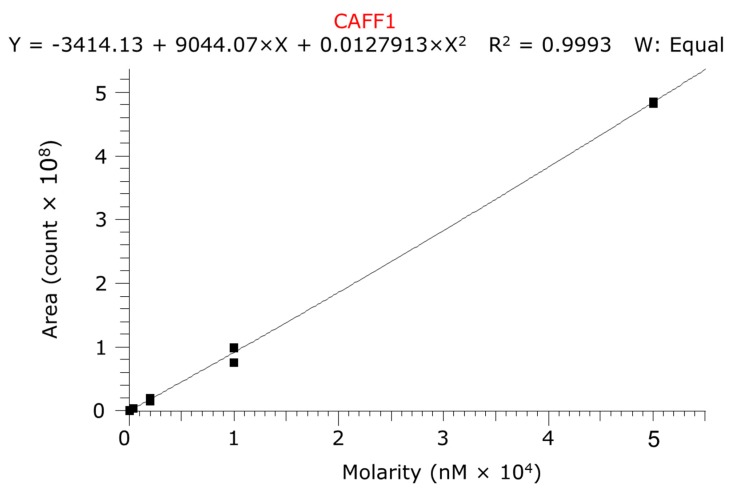
Calibration curve of caffeine. The fitting was quadratic model without weighting.

The quantitative LC-MS/MS analysis was done in positive electrospray ionization (ESI) mode on Thermo Q Exactive Focus orbital mass spectrometer coupled with Thermo Dionex Ultimate 3000 UHPLC system (UNICAM Hungary Ltd., Budapest, Hungary). The high performance liquid chromatography (HPLC) column was Phenomenex Kinetex XB-C18, 2.1 × 50 mm column (Gen-Lab Ltd., Budapest, Hungary) used in isocratic chromatographic conditions where the eluent was containing 8% acetonitrile and 0.1% formic acid in water. The analysis time was 1.5 min. The mass spectrometer operated in PRM (Parallel Reaction Monitoring) mode where *m*/*z*: 195.0876 parent ion was fragmented at NCE: 55 (scan range: 50.0000–220.0000 *m*/*z*). The *m*/*z*: 138.06606 ion was used as a quantifier ion. The ESI source parameters were the following: Spray voltage (V): 3500, capillary temperature (°C): 256, probe heater temperature (°C): 413, sheath gas: 48, auxiliary gas: 11, sweep gas: 2, all gas flow rates are in arbitrary units. A typical chromatogram of caffeine is presented in Figure 4. Five calibration levels were used by the quantitation in 0.08–50 µM range with 1:5 dilution pattern (Figure 5).

### 2.7. Histology

One-one representative skin preparation was taken from a native mouse, a native rat, a sensitized (10 tape-stripping) mouse and a sensitized (10 tape-stripping) rat. The skin samples were placed into 10% formaldehyde and paraffin, and embedded. To analyze the cellular changes caused by the sensitization process Hematoxylin-Eosin staining was used.

## 3. Results

### 3.1. Franz Diffusion Cell Studies

**Figure 6:**
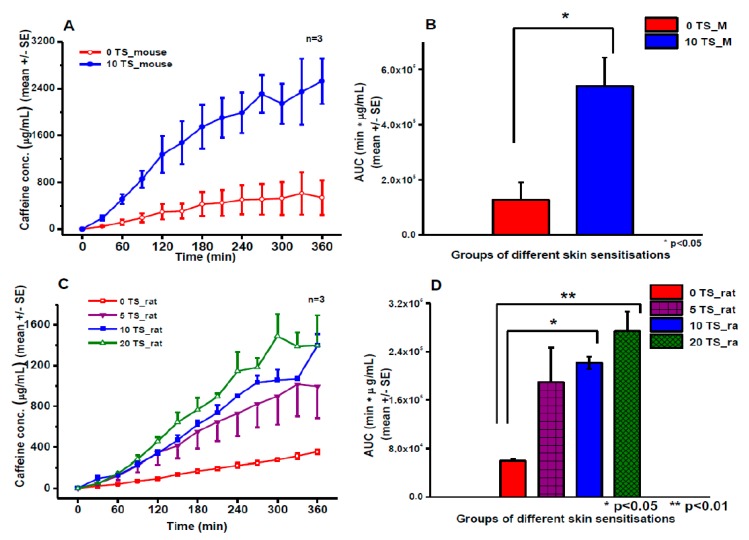
Transdermal caffeine absorption from a cream formulation studied in Franz diffusion cells. Concentration-time profiles of caffeine absorption in mouse (**A**) and rat (**C**) skin preparations. Area under the curves (AUCs) of concentration-time profiles in mouse (**B**) and in rat (**D**). 0TS: native skin without tape-stripping, 5-10-20TS: sensitized skin with 5-10-20 tape-strippings. *N* = 3, *: *p* < 0.05, **: *p* < 0.01 in two-sample student *t*-test.

In the ex vivo Franz cell experiments non-sensitized and sensitized mouse and rat skins were compared for caffeine permeability (Figure 6). Both in rats and in mice statistically significant differences have been detected after the 10-tape-stripping (10 TS) in the caffeine absorption compared to the respective controls (0 TS). The degree of caffeine penetration correlated closely with the number of sensitizations in rats. As the permeability of the mouse skin was higher in non-sensitized animals than that of in rats (*AUC*_M0TS_ = 127,466 ± 63,424 vs. *AUC*_R0TS_ = 58,823 ± 3933 μg/mL·min) it is not surprising that the effect of the 10 tape-stripping was also more pregnant in mice than in rats (*AUC*_M10TS_ = 540,196 ± 102,528 vs. *AUC*_R10TS_ = 221,358 ± 10,394 μg/mL·min).

### 3.2. Transdermal Microdialysis Study

**Figure 7:**
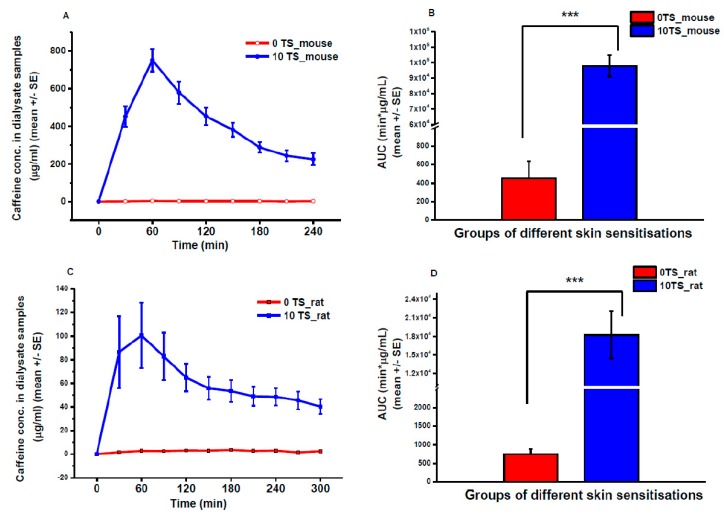
Caffeine absorption in vivo in anesthetized rodents measured by transdermal microdialysis. Concentration-time profiles of caffeine skin penetration in mouse (**A**) and rat (**C**) abdominal skin surface. Area under the concentration-time curves (AUC) in mice (**B**) and in rats (**D**). 0 TS: native skin, without tape-stripping; 10 TS: sensitized skin, with 10 tape-strippings. *N* = 5, ***: *p* < 0.005 in two-sample student *t*-test.

Caffeine absorption was also compared in vivo in mice and rats with and without skin sensitization (Figure 7). The free levels of caffeine in the dermis showed a peak in both species at 60 min after the placement of caffeine cream containing patches on the skin. However, the *C*_max_ values were about 8-fold higher in mice than in rats. The elimination dynamics of the drug showed faster characteristics in the mouse than in the rat. The in vivo data are in line with the ex vivo Franz diffusion cell results concerning the degree and the statistical strength (significance) of the effect of tape-stripping and regarding the difference between the two species (*AUC*_M0TS_ = 452 ± 202 vs. AUC_M10TS_ = 77,167 ± 34,510 μg/mL·min and *AUC*_R0TS_ = 741 ± 234 vs. *AUC*_R10TS_ = 18,222 ± 5762 μg/mL·min).

### 3.3. Skin-on-a-Chip Diffusion Cell Study

**Figure 8:**
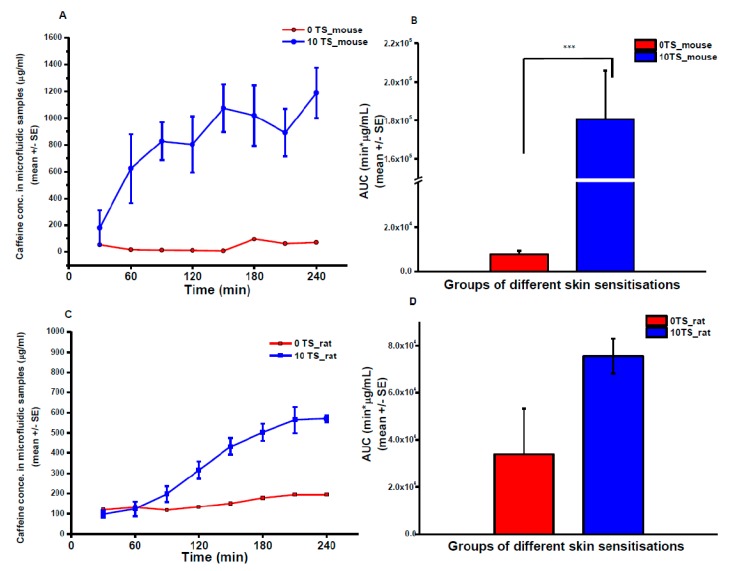
Caffeine absorption in rodent skin preparations measured by skin-on-a-chip device (MDC). Concentration-time profiles of caffeine skin penetration in mouse (**A**) and rat (**C**) abdominal skin preparations. Area under the concentration-time curves (AUC) in mice (**B**) and in rats (**D**). 0 TS: native skin, without tape-stripping; 10 TS: sensitized skin, with 10 tape-strippings. *N* = 3, ***: *p* < 0.005 in two-sample student *t*-test.

Caffeine absorption was measured also in Microfluidic Diffusion Chamber (MDC) (Figure 8). The degree of absorption was approximately 50% of that measured by Franz cell. However, it is important to mention that the caffeine content of Franz cell samples was determined by spectroscopy, while the microfluidic samples were analyzed by LC-MS/MS. Based on these methodological differences the results can only be evaluated separately. In the chip, similarly to microdialysis data, the native rat skin was more permeable than mouse skin (*AUC*_M0TS_ = 7902 ± 1402 vs. *AUC*_R0TS_ = 15,900 μg/mL·min), but the sensitization resulted in higher increase in caffeine absorption in mouse skin than in rat skin (*AUC*_M10TS_ = 180,621 ± 25,217 and *AUC*_R10TS_ = 75,588 ± 7389 μg/mL·min).

### 3.4. Histology

**Figure 9:**
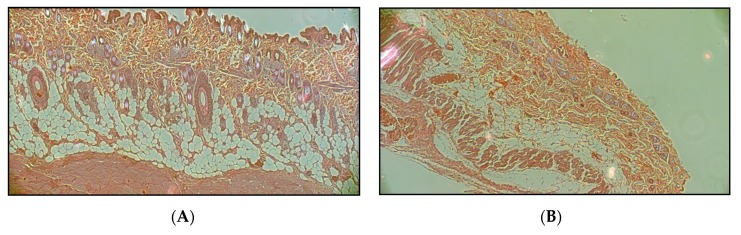

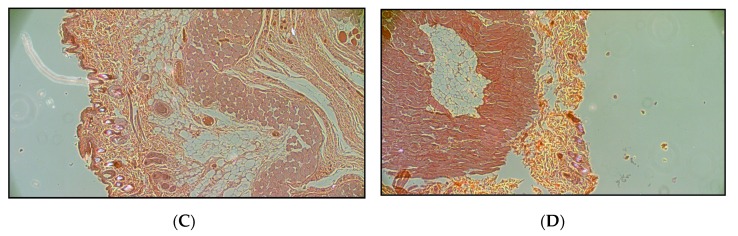
Histological structure of native and sensitized rat and mouse abdominal skin preparations (Hematoxylin-Eosin staining). (**A**): native rat skin (0 tape-stripping), (**B**): native mouse skin (0 tape-stripping), (**C**): sensitized (10 tape-strippings) rat skin, (**D**): sensitized (10 tape-strippings) mouse skin.

Figure 9 shows that the tape-stripping more or less removed the external surface of stratum corneum in the sensitized skins (arrows in Figure 9C,D).

## 4. Discussion and Conclusions

Many compounds are applied on the skin either deliberately or accidentally, with either beneficial or deleterious outcomes. The dermal absorption assessment can be important for evaluating: (a) local effects in dermatology (e.g., corticosteroids for dermatitis); (b) delivery through the skin seeking a systemic effect (e.g., nicotine patches, hormonal drug patches, etc.); (c) surface effects (e.g., sunscreens, cosmetics, and anti-infectives) [18,19]; (d) targeting of deeper tissues (e.g., non-steroidal anti-inflammatory agents) [20,21,22,23,24,25,26,27,28,29]; and (e) unwanted absorption (e.g., solvents in the workplace, pesticides or allergens) [19,30,31].

Removal of the outermost skin layer of stratum corneum by tape-stripping has become a common practice in recent decades for preclinical testing [19,32,33]. The determination of the kinetics and penetration depth of different drugs can be facilitated using the practically non-invasive method of tape-stripping with adhesive tape [34,35]. Tape-stripping makes easier the evaluation of bioequivalence of topical dermatological dosage forms and makes possible to reach detectable concentrations of the drugs in the dermis and subcutis in preclinical animal models [4,36]. Therefore, in our study, tape-stripped and non-stripped skins were studied in parallel for permeability.

For the investigation of skin penetration of drugs, unwanted chemicals, and cosmetics, new techniques are needed which can use the knowledge gained from the previous in vitro, ex vivo, and in vivo studies, but require fewer biomaterials, skins, skin equivalents, and test compounds. For this reason, the microfluidic environment looks an optimal approach to reach our goal: to miniaturize the diffusion set-up. After some previous studies in cell culture platforms [12,37,38] to realize this approach, our laboratory produced a device to fulfill the requirements of the pharmaceutical and cosmetic industry to make an economical and reliable drug-screening platform. The parallelization, automation, and further developments according to the requirements of the researchers and end users are in progress.

In the current study, the first biological evaluation of a novel MDC device is presented using the most easily available skin preparations from rodents. This assay can be considered to be a first level of ex vivo screening for topical drugs. The investigation of drugs can be further continued by artificial skin equivalents or even human skins.

In comparison with data gained from traditional Franz cell experiments, our MDC device seems to provide similar information about the characteristics, degree, and speed of absorption of the test drug (caffeine) through the dermal barrier. Although the *C*_max_ values show an approximately two- fold difference between the Franz cell and the microfluidic data, this variability might have different backgrounds. One possible explanation is the different bioanalytical methods used (as mentioned earlier), and the other possibility is the material of the acceptor module of the microfluidic chamber which was made of PDMS. This polymer has a strong absorptive property for small molecules, mainly for lipophilic compounds [39,40]. Although caffeine is a very hydrophilic molecule (log*P* = −0.79), it can be assumed that there is some non-specific interaction with the chamber’s material. Therefore, the next step in the validation of the device will be the application of bovine serum albumin (BSA) in the perfusion fluid to reduce the possible non-specific binding to PDMS. The other option is to change the material of the acceptor panel e.g., to glass or other less absorptive material.

Recently, several experiments have been performed worldwide to use skin equivalents in the drug penetration experiments [41,42,43,44,45]. To develop more sophisticated skin substituents and optimize them for possible dermatological, pharmaceutical, or surgical purposes, our device would provide a reliable, low-cost testing platform.

In conclusion, in this paper a new skin-on-a chip microfluidic device has been presented. It was tested and compared with traditional drug penetration assays and optimized for pharmaceutical purposes. It can be further developed for special use, e.g., for testing cell culture platforms, artificial skins, or other tissues such as oral or nasal mucosa, or eye formulations. By further engineering, the parallelization of the device can also be implemented.

## References

[1] Leveque N., Makki S., Hadgraft J., Humbert P. (2004). Comparison of Franz cells and microdialysis for assessing salicylic acid penetration through human skin. Int. J. Pharm..

[2] Iadaresta F., Manniello M.D., Östman C., Crescenzi C., Holmbäck J., Russo P. (2018). Chemicals from textiles to skin: An *in vitro* permeation study of benzothiazole. Environ. Sci. Pollut. Res. Int..

[3] Haq A., Goodyear B., Ameen D., Joshi V., Michniak-Kohn B. (2018). Strat-M^®^ synthetic membrane: Permeability comparison to human cadaver skin. Int. J. Pharm..

[4] Farner F., Bors L., Bajza Á., Karvaly G., Antal I., Erdő F. (2019). Validation of an In vitro-in vivo Assay System for Evaluation of Transdermal Delivery of Caffeine. Drug Deliv. Lett..

[5] Frosini S.M., Bond R., Loeffler A., Larner J. (2017). Opportunities for topical antimicrobial therapy: Permeation of canine skin by fusidic acid. BMC Vet. Res..

[6] Roy S.D., de Groot J.S. (1994). Percutaneous absorption of nafarelin acetate, an LHRH analog, through human cadaver skin and monkey skin. Int. J. Pharm..

[7] Taofiq O., Rodrigues F., Barros L., Barreiro M.F., Ferreira I.C.F.R., Oliveira M.B.P.P. (2019). Mushroom ethanolic extracts as cosmeceuticals ingredients: Safety and ex vivo skin permeation studies. Food Chem. Toxicol..

[8] Berkó S., Zsikó S., Deák G., Gácsi A., Kovács A., Budai-Szűcs M., Pajor L., Bajory Z., Csányi E. (2018). Papaverine hydrochloride containing nanostructured lyotropic liquid crystal formulation as a potential drug delivery system for the treatment of erectile dysfunction. Drug Des. Dev. Ther..

[9] Haq A., Michniak-Kohn B. (2018). Effects of solvents and penetration enhancers on transdermal delivery of thymoquinone: Permeability and skin deposition study. Drug Deliv..

[10] Bal-Öztürk A., Miccoli B., Avci-Adali M., Mogtader F., Sharifi F., Çeçen B., Yaşayan G., Braeken D., Alarcin E. (2018). Current Strategies and Future Perspectives of Skin-on-a-Chip Platforms: Innovations, Technical Challenges and Commercial Outlook. Curr. Pharm. Des..

[11] Alexander F.A., Eggert S., Wiest J. (2018). Skin-on-a-Chip: Transepithelial Electrical Resistance and Extracellular Acidification Measurements through an Automated Air-Liquid Interface. Genes.

[12] Alberti M., Dancik Y., Sriram G., Wu B., Teo Y.L., Feng Z., Bigliardi-Qi M., Wu R.G., Wang Z.P., Bigliardi P.L. (2017). Multi-chamber microfluidic platform for high-precision skin permeation testing. Lab Chip.

[13] van den Broek L.J., Bergers L.I.J.C., Reijnders C.M.A., Gibbs S. (2017). Progress and Future Prospectives in Skin-on-Chip Development with Emphasis on the use of Different Cell Types and Technical Challenges. Stem Cell Rev. Rep..

[14] Mori N., Morimoto Y., Takeuchi S. (2017). Skin integrated with perfusable vascular channels on a chip. Biomaterials.

[15] Mohammadi M.H., Heidary Araghi B., Beydaghi V., Geraili A., Moradi F., Jafari P., Janmaleki M., Valente K.P., Akbari M., Sanati-Nezhad A. (2016). Skin Diseases Modeling using Combined Tissue Engineering and Microfluidic Technologies. Adv. Healthc. Mater..

[16] Erdő F. (2015). Microdialysis Techniques in Pharmacokinetic and Biomarker Studies. Past, Present and Future Directions. A Review. Clin. Exp. Pharmacol..

[17] Erdő F., Hashimoto N., Karvaly G., Nakamichi N., Kato Y. (2016). Critical evaluation and methodological positioning of the transdermal microdialysis technique. A review. J. Control. Release.

[18] Olvera-Martínez B.I., Cazares-Delgadillo J., Calderilla-Fajardo S.B., Villalobos-García R., Ganem-Quintanar A., Quintanar-Guerrero D. (2005). Preparation of polymeric nanocapsules containing octyl methoxycinnamate by the emulsification–diffusion technique: Penetration across the stratum corneum. J. Pharm. Sci..

[19] Escobar-Chávez J.J., Merino-Sanjuán V., López-Cervantes M., Urban-Morlan Z., Piñón-Segundo E., Quintanar-Guerrero D., Ganem-Quintanar A. (2008). The tape-stripping technique as a method for drug quantification in skin. J. Pharm. Pharm. Sci..

[20] Escobar-Chávez J.J., López-Cervantes M., Naïk A., Kalia Y.N., Quintanar-Guerrero D., Ganem Quintanar A. (2006). Applications of the thermoreversible Pluronic F-127 gels in pharmaceutical formulations. J. Pharm. Pharm. Sci..

[21] Miyazaki S., Yokouchi C., Nakamura T., Hashiguchi N., Hou W.M., Takada M. (1986). Pluronic F-127 gels as a novel vehicle for rectal administration of indomethacin. Chem. Pharm. Bull..

[22] Chi S.C., Tan H.K., Chun H.W. (1996). Antiinflammatory and Analgesic Transdermal Gel. U.S. Patent.

[23] Fang J.Y., Leu Y.L., Wang Y.Y., Tsai Y.H. (2002). In vitro topical application and in vivo phramacodynamic evaluation of nonivamide hydrogels using Wistar rat as an animal model. Eur. J. Pharm. Sci..

[24] Shin S.C., Cho C.W., Oh I.J. (2001). Effects of non ionic surfactants as permeation enhancers towards piroxicam from the poloxamer gel through rat skins. Int. J. Pharm..

[25] Liaw J., Lin Y.C. (2000). Evaluation of poly(ethylene oxide)–poly(propylene oxide)–poly(ethylene oxide) (PEO–PPO–PEO) gels as a release vehicle for percutaneous fentanyl. J. Control. Release.

[26] Wang Y.Y., Hong C.T., Chiu W.T., Fang J.Y. (2001). In vitro and in vivo evaluations of topically applied capsaicin and nonivamide from hydrogels. Int. J. Pharm..

[27] El-Kattan A.F., Asbill C.S., Kim N., Michniak B.B. (2000). Effect of formulation variables on the percutaneous permeation of ketoprofen from gel formulations. Drug Deliv..

[28] Curdy C., Kalia Y.N., Naïk A., Guy R.H. (2001). Piroxicam delivery into human stratum corneum in vivo: Iontophoresis versus passive diffusion. J. Control. Release.

[29] Escobar-Chávez J.J., Quintanar-Guerrero D., Ganem-Quintanar A. (2005). In vivo skin permeation of sodium naproxen formulated in PF-127 gels: Effect of Azone^®^ and Transcutol^®^. Drug Dev. Ind. Pharm..

[30] Mattorano D.A., Kupper L.L., Nylander-French L.A. (2004). Estimating dermal exposure to jet fuel (naphthalene) using adhesive tape strip samples. Ann. Occup. Hyg..

[31] Chao Y.C., Nylander-French L.A. (2004). Determination of Keratin Protein in a Tape stripped Skin Sample from Jet Fuel Exposed Skin. Ann. Occup. Hyg..

[32] Surakka J., Lindh T., Rosen G. (2000). Workers’ dermal exposure to UV-curable acrylates in the furniture and parquet industry. Ann. Occup. Hyg..

[33] Nylander-French L.A. (2000). A tape-stripping method for measuring dermal exposure to multifunctional acrylates. Ann. Occup. Hyg..

[34] Moser K., Kriwet K., Naik A., Kalia Y.N., Guy R.H. (2001). Passive skin penetration enhancement and its quantification in vitro. Eur. J. Pharm. Biopharm..

[35] Pinkus H. (1966). Tape stripping in dermatological research. A review with emphasis on epidermal biology. Giormale Ital. Derm. Minerva Dermatol..

[36] Löffler H., Dreher F., Maibach H.I. (2004). Stratum corneum adhesive tape stripping: Influence of anatomical site, application pressure, duration and removal. Br. J. Dermatol..

[37] Mattern K., Beißner N., Reichl S., Dietzel A. (2018). DynaMiTES—A dynamic cell culture platform for in vitro drug testing PART 1—Engineering of microfluidic system and technical simulations. Eur. J. Pharm. Biopharm..

[38] Beiβner N., Mattern K., Dietzel A., Reichl S. (2018). DynaMiTES—A dynamic cell culture platform for in vitro drug testing PART 2—Ocular DynaMiTES for drug absorption studies of the anterior eye. Eur. J. Pharm. Biopharm..

[39] Toepke M.W., Beebe D.J. (2006). PDMS absorption of small molecules and consequences in microfluidic applications. Lab Chip.

[40] Li N., Schwartz M., Ionescu-Zanetti C. (2009). PDMS Compound Adsorption in Context. J. Biomol. Screen..

[41] Hönzke S., Wallmeyer L., Ostrowski A., Radbruch M., Mundhenk L., Schäfer-Korting M., Hedtrich S. (2016). Influence of Th2 Cytokines on the Cornified Envelope, Tight Junction Proteins, and ß-Defensins in Filaggrin-Deficient Skin Equivalents. J. Investig. Dermatol..

[42] Petrova A., Celli A., Jacquet L., Dafou D., Crumrine D., Hupe M., Arno M., Hobbs C., Cvoro A., Karagiannis P. (2014). 3D in vitro model of a functional epidermal permeability barrier from human embryonic stem cells and induced pluripotent stem cells. Stem. Cell Rep..

[43] van Drongelen V., Danso M.O., Mulder A., Mieremet A., van Smeden J., Bouwstra J.A., El Ghalbzouri A. (2014). Barrier properties of an N/TERT-based human skin equivalent. Tissue Eng. Part A.

[44] Wagner H., Kostka K.H., Lehr C.M., Schaefer U.F. (2001). Interrelation of permeation and penetration parameters obtained from in vitro experiments with human skin and skin equivalents. J. Control. Release.

[45] Zghoul N., Fuchs R., Lehr C.M., Schaefer U.F. (2001). Reconstructed skin equivalents for assessing percutaneous drug absorption from pharmaceutical formulations. Altex.

